# Carbonic Anhydrases: A Superfamily of Ubiquitous Enzymes

**DOI:** 10.3390/ijms24087014

**Published:** 2023-04-10

**Authors:** Clemente Capasso

**Affiliations:** Institute of Biosciences and Bioresources, National Research Council (CNR), via Pietro Castellino 111, 80131 Napoli, Italy; clemente.capasso@ibbr.cnr.it

Numerous physiological and pathological cellular processes depend on the ability to detect and respond to changes in gas levels both inside and outside the cell [1]. Overall, the ability of cells to detect and respond to changes in gas levels is critical for maintaining homeostasis and ensuring proper cellular function. The gas-sensing requires a variety of reactions, involving metal centers of metalloproteins or thiol modification of protein cysteine residues [1]. These reactions/modifications generate signaling cascades, which play a vital role in alterations of metabolic systems in physiologic and pathologic conditions.

CO_2_ is released into the atmosphere by cellular respiration and oxidative metabolism, and all living organisms, from bacteria to humans, produce it [2]. CO_2_ is not just a waste product, as it can activate multiple cellular signaling pathways and be used as C1 source of carbon in biosynthetic reactions. Changes in CO_2_ levels influence mammalian cellular function through modulation of signal transduction networks influenced by pH, mitochondrial function, adenylate cyclase sensing, calcium signaling, transcriptional regulators, etc. [3]. CO_2_ has also been found to act as a signaling molecule in plant cells, where it can regulate stomatal closure and photosynthesis [4]. Opportunistic and pathogenic fungi sense the CO_2_ difference, which influences their differentiation, determining the expression of those fungal features essential for virulent or non-virulent phenotypes [5,6]. Bacteria, such as *Vibrio cholerae*, produce enterotoxin when CO_2_ levels rise, while bicarbonate produced by CO_2_ hydration enhances the ability of the gene transcription activator (ToxT) to activate the production of cholera toxin (CT) [3,7]. *Pseudomonas aeruginosa*, which can lead to infections in the blood, lungs (pneumonia), or other regions of the body following surgery, lives in vastly varying CO_2_ settings depending on whether or not it is colonizing a host [8].

Biomolecules related to CO_2_-sensitive pathways or acting as CO_2_ transducers have been proposed as appealing targets for medicines since they control cell development and several subsequent biosynthetic pathways [5,6]. In this context, a crucial role is played by a superfamily of proteins known as carbonic anhydrases (CAs, EC 4.2.1.1), which catalyze the reversible hydration of CO_2_, converting it into bicarbonate and protons and adjust the CO_2_/bicarbonate levels in response to environmental changes or physiological demands [3,5,6,9].

This Special Issue of *IJMS* presents a topical collection of articles dealing with CAs from mammals, bacteria, fungi, and microalgae. The bacterial CAs play a crucial role in making the hydration–dehydration reaction faster, guaranteeing the microbial metabolic demands and its survival [9]. In Gram-negative bacteria, for example, CAs localized in the periplasmic space convert the metabolic and atmospheric CO_2_ into bicarbonate, to prevent CO_2_ leakage, whereas the cytoplasmic CAs provide CO_2_ and bicarbonate to the central bacterial metabolism [10,11,12,13]. In fungi, two molecules are crucial for fungal CO_2_-sensing: (1) bicarbonate (HCO_3_^−^), which is a meiosis- and sporulation-promoting ion, and (2) adenylyl cyclase (AC) that is involved in the spore formation. The fungal CO_2_-sensing, related to the CO_2_/HCO_3_^−^/pH-sensing system, is directly stimulated by HCO_3_^−^ produced in a CA-dependent manner [5,6]. In mammals, CA activity helps to maintain the acid–base balance in the body and some CA isoforms are involved in tumor cell motility [14,15]. For example, the human isoform CA (hCAIX) expression is usually induced by hypoxia in certain types of solid tumors, such as glioma, breast cancer, and colon carcinoma [16]. In microalgae, CAs play important roles in carbon concentration mechanisms (CCMs) that enable these organisms to efficiently fix CO_2_ for photosynthesis, even under conditions of limited CO_2_ availability [17].

The existence of a link between CAs and microorganism metabolism represents a challenge in discovering antibacterials, especially in the era of antibiotic resistance [18]. In this Special Issue, Akdemir and coworkers demonstrated that selective inhibition of the α-class CA from the pathogenic bacterium *Vibrio cholerae* (VcCA) with a series of hydrazone derivatives, carrying the 2-(hydrazinocarbonyl)-3-phenyl-1*H*-indole-5-sulfonamide scaffold, presents an alternative therapeutic target to contrast the acute diarrheal infection provoked by *Vibrio cholera* [19]. These groups also tested 26 thiazolidinones against the *Candida glabrata* β-CA (CgNce103) and the *Candida* spp., demonstrating that these compounds had selective antifungal activity with a potency like fluconazole and clotrimazole [20]. Capasso and Supuran cloned, expressed, purified and characterized the β-CA (MreCA) from the genome of the pathogenic fungus *M. restricta*, responsible for dandruff, together with other fungi and bacteria. MreCA resulted in high catalytic activity for the hydration of CO_2_ into bicarbonate and protons and was sensitive to inhibition by the classical sulfonamide inhibitor acetazolamide (K_I_ of 50.7 nM) [21]. It is also the contribution of Aspatwar of the diseases caused by *Mycobacterium tuberculosis * [22]. They investigate the biochemical and molecular characterization of β-CAs encoded by the genome of this microorganism, discovering that this enzyme is implicated in several nonenzymatic functions that are required for the survival of *M. tuberculosis* and pathogenesis of tuberculosis disease in the host. It is the review presented by Gontero on the diversity of CAs and their roles in microalgae, focalizing their attention on the carbon dioxide-concentrating mechanisms and photosynthesis, their regulation, as well as their less studied roles in non-photosynthetic processes [23].

In the literature, it has been reported that the abnormal expression of one or multiple CA isoforms in humans usually is associated to various pathologies. Thus, the use of proper CA modulators (i.e., traditionally CA inhibitors, CAIs) represented an interesting strategy for the management of various diseases such as glaucoma, altitude sickness, epilepsy, and obesity. In this context, Supuran’s group prepared a variety of compounds characterized by a substitution pattern on the ring and the tails, located on para- or meta-positions, obtaining benzylaminoethyureido-tailed benzenesulfonamides [24]. Most of these new compounds showed low nM inhibitors when tested against human carbonic anhydrases (hCA) isoforms I, II, IX, and XII, involving various pathologies. Eldehna et al. presented the synthesis and biological evaluation of novel series of diamide-based benzenesulfonamide compounds as inhibitors of the isoforms hCA I, II, IX, and XII [25]. Mannelli’s group tested in a rat model of rheumatoid arthritis new dual inhibitors incorporating both a carbonic anhydrase (CA)-binding moiety and a cyclooxygenase inhibitor (NSAID) [26]. This new class of potent molecules showed a high activity in the preclinical condition of rheumatoid arthritis, suggesting a novel attractive pharmacodynamic profile. Lakota and colleagues presented an article which investigated the role of the isoform hCA I and its mechanism of action in spontaneous tumor regression [27]. Winum and coworkers used the multivalency approach to design CA inhibitors belonging to a variety of classes (sulfonamides, dithiocarbamates, carboxylates, etc.) with the scope to enhance the binding affinity and selectivity versus the mammalian or bacterial CAs [28]. Fossati’s group presented an excellent review on the preclinical and clinical findings regarding the role of CAs and their inhibitors/activators on cognition, aging, and neurodegeneration, discussing the future challenges and opportunities in the field [29]. Emameh and coworkers presented a fascinating review describing the state-of-the-art potential of phytochemicals as modulators of long non-coding RNAs, a group of transcripts involved in various biological processes, and the overexpression of some tumor-associated proteins, including CA II, CA IX, and CA XII [30]. Moreover, it has been reported in the literature that CA VI is a protein that is secreted into milk and saliva, and it can act as a trophic element in the healing process of wounds in animals that typically lick their wounds. Järvinen’s group presented a manuscript in which demonstrated that CA VI does not significantly affect skin-wound healing [31].

Finally, carbon dioxide is considered one of the major causes of climate change, by its accumulation in the atmosphere and its greenhouse effects. The search for environmentally friendly methodologies to directly sequester CO_2_ from the production sites is actively supported. Since the slowest step of hydration is enhanced by carbonic anhydrase, the biomimetic approach, based on the use of innovative CAs as a biocatalyst, results in an excellent strategy in terms of eco-compatibility and reduction of the energy penalty. Moreover, the biomimetic approach represents a pivotal point for the research and development of new strategies for the CO_2_ conversion pathways, which should be developed now to prepare for a rapidly changing future. In this field, Crespo’s group demonstrated that low CA concentrations (0.2 mg g^−1^) proved to be sufficient to improve the overall CO_2_ capture process performance [32].

Overall, the study of CAs, which are biomolecules involved in CO_2_-sensitive pathways or act as CO_2_ transducers, represents a promising avenue for developing new therapeutic strategies targeting a wide range of infections and diseases as well as represent a new approach for CO_2_ conversion, mitigating the effects of climate change.
